# Validation of anaemia, haemorrhage and blood disorder reporting in hospital data in New South Wales, Australia

**DOI:** 10.1186/s13104-021-05584-x

**Published:** 2021-05-04

**Authors:** Heather J. Baldwin, Tanya A. Nippita, Siranda Torvaldsen, Therese M. McGee, Kristen Rickard, Jillian A. Patterson

**Affiliations:** 1 The University of Sydney Northern Clinical School, Women and Babies Research, C/O University Department of O&G, Level 5, Douglas Building, Royal North Shore Hospital, St Leonards, NSW 2065 Australia; 2 Northern Sydney Local Health District, Kolling Institute, St Leonards, NSW Australia; 3 Department of Obstetrics and Gynaecology, Royal North Shore Hospital, St Leonards, NSW Australia; 4 School of Public Health and Community Medicine, UNSW, Sydney, NSW Australia; 5 OG Department, Clinical Support Unit, Level 3, G Block, Westmead Hospital, Westmead, NSW Australia; 6 The University of Sydney Westmead Clinical School, Westmead, NSW Australia

**Keywords:** Validity, Anaemia, Haemorrhage, Blood disorders, Platelet disorders, Coagulation disorders, Transfusion, Hysterectomy

## Abstract

**Objective:**

Hospital data are a useful resource for studying pregnancy complications, including bleeding-related conditions, however, the reliability of these data is unclear. This study aims to examine reliability of reporting of bleeding-related conditions, including anaemia, obstetric haemorrhage and blood disorders, and procedures, such as blood transfusion and hysterectomy, in coded hospital records compared with obstetric data from two large tertiary hospitals in New South Wales.

**Results:**

There were 36,051 births between 2011 and 2015 included in the analysis. Anaemia and blood disorders were poorly reported in the hospital data, with sensitivity ranging from 2.5% to 24.8% (positive predictive value (PPV) 12.0–82.6%). Reporting of postpartum haemorrhage, transfusion and hysterectomy showed high sensitivity (82.8–96.0%, PPV 78.0–89.6%) while moderate consistency with the obstetric data was observed for other types of obstetric haemorrhage (sensitivity: 41.9–65.1%, PPV: 50.0–56.8%) and placental complications (sensitivity: 68.2–81.3%, PPV: 20.3–72.3%). Our findings suggest that hospital data may be a reliable source of information on postpartum haemorrhage, transfusion and hysterectomy. However, they highlight the need for caution for studies of anaemia and blood disorders, given high rates of uncoded and ‘false’ cases, and suggest that other sources of data should be sought where possible.

**Supplementary Information:**

The online version contains supplementary material available at 10.1186/s13104-021-05584-x.

## Introduction

Hospital data are an efficient, cost-effective resource to study trends and outcomes of medical conditions and procedures [1–5], including during pregnancy. The range of procedures and conditions that can affect pregnant women and their babies are not always captured in obstetric data. Obstetric data are often recorded in a form difficult to analyse, such as free text, and are less commonly available on a population level than hospital data. Hospital data, comprised of coded diagnoses and procedures following international standards, provide a useful alternative or supplement to obstetric data for the purposes of research [1–6]. However, the reliability of findings depends on the extent to which hospital data accurately identify patients with and without the condition or procedure. Discrepancies in hospital data can occur at various stages of the recording process, including initial documentation, coding, and data entry, and accuracy may change over time, influenced by changes in practice, guidelines, and focus on particular conditions.

Anaemia, obstetric haemorrhage, blood disorders such as coagulation and platelet disorders, and related procedures, are associated with adverse maternal and neonatal outcomes [7–10]. Previously, nutritional anaemia [11], placental abruption [12, 13] and hysterectomy [13] have been shown to have poor sensitivities, while haemolytic anaemia [11], transfusion [13], and coagulation disorders [11] have been shown to have good sensitivities in hospital data. However, these studies were based on births in 2000 [12] and 2002 [11, 13], and it is not known whether reporting has changed in the intervening years.

Validation studies comparing coded hospital data with medical charts can be used to assess the reliability of data sources, but are time consuming, expensive, and tend to review a small number of records and short time period. An alternative approach is to compare two independent databases [14–16]. Here, we compare reporting of bleeding-related conditions and procedures in pregnancy in coded hospital data extracted from the electronic medical record to obstetric data from the ObstetriX database, using ObstetriX as the reference standard.

The hospital data are coded following the International Classification of Diseases (ICD) and Australian Classification of Health Interventions by trained clinical coders, using clinical documentation during an inpatient episode of care to assign the appropriate diagnosis, and where relevant, procedure code(s). Government policy and ICD coding standards mostly limit what is coded to conditions affecting the current admission, require substantiation by clear medical record documentation, and prohibit interpretation of results [17]. Coded data are used to facilitate activity based funding, healthcare management and planning, and also inform the population-level New South Wales Admitted Patient Data Collection.

ObstetriX is a clinical database specific to the pregnancy, birth and early postnatal period, collected by midwives, a subset of which forms the statewide New South Wales Perinatal Data Collection. This population-based data collection has shown high levels of accuracy for reporting of diagnoses and procedures during labour and delivery [18], and validation studies of similar Australian data collections such as the Victorian Perinatal Data Collection have shown high accuracy for most data items [19–21]. Given the different purposes and perspectives of the databases, ObstetriX may be considered an imperfect reference standard, however most reference standards are not without error and uncertainty, particularly where self-report is relied upon [22–24], and large population datasets have been shown to be robust to the introduction of random errors and omissions [25].

The aim of this study is to determine the consistency of reporting of anaemia, haemorrhage and blood disorders during pregnancy, and related procedures including blood transfusion and hysterectomy, in hospital records compared with an obstetric database. We aimed to compare reporting between two large tertiary hospitals, and to determine whether patient or pregnancy characteristics affect reporting.

## Main text

### Methods

Women giving birth to singleton infants (≥ 24 weeks gestation) in two tertiary hospitals in the Sydney metropolitan area, New South Wales (NSW), Australia, between 2011 and 2015 were included. In NSW, all births in a hospital or birth centre are treated as inpatient admissions and assigned an electronic medical record and an obstetric record. Delivery in hospital and birth centres represent 99% of births in the state [26]. Women who had prearranged to give birth in a different hospital to the hospital of birth were excluded, because antenatal data would have been collected at the hospital of booking and may be incomplete.

ObstetriX contains maternal health and demographic data, obstetric history and pregnancy details. Initial information is obtained on pregnancy and medical history at the face-to-face booking consultation with a midwife (an outpatient encounter, by 16 weeks gestation). The record is updated with labour, birth and postnatal information collected during the birth admission. Data are entered by midwives and recorded in checkboxes or drop-down menus, with a small amount of free text available. The majority of procedures and conditions are recorded as present, absent or unknown/missing. The midwives do not have access to the hospital codes, as coding is performed four to six weeks after discharge.

The coded hospital data contain diagnoses coded according to the International Classification of Diseases, Tenth Revision, Australian Modification (ICD-10-AM) with a small number coded using Systematized Nomenclature of Medicine—Clinical Terms (SNOMED CT) codes. Procedures are coded following the Australian Classification of Health Interventions, Eighth Edition. Coding is performed by trained clinical coders based on clinical documentation in the electronic medical record. Medical records for admissions throughout pregnancy and the birth were searched for the relevant diagnoses and procedures (codes provided in Additional file 1: Table S1).

Records were deterministically linked using patient Medical Record Numbers and checked using other personal identifiers, by personnel external to the project. Data were de-identified data prior to analysis.

Reporting in hospital data was compared to that in obstetric data as the reference standard. Sensitivity, specificity, positive predictive values (PPV) and negative predictive values (NPV) are reported with exact confidence intervals. These measures were also calculated separately for the two hospitals. Analyses were performed in SAS 9.3.

## Results

Of the 38,343 singleton births of at least 24 weeks gestation between 2011 and 2015, 36,051 (95.1%) gave birth at their booking hospital and were included in the analysis.

Sensitivities ranged from very low (2.5%, thalassaemia) to high (96.0%, hysterectomy) (Table 1). Sensitivity and PPV for anaemia were poor (sensitivity: 4.4–16.4%, PPV: 12.0–38.1%). Sensitivity for obstetric haemorrhage ranged from 56.3 to 89.7%, with PPV ranging from 50.0 to 91.8%. For blood disorders, sensitivity ranged from 2.5–24.8% and PPV from 15.3–82.6%, and for placental complications sensitivity ranged from 68.2 to 81.3% and PPV from 20.3 to 72.3%. Specificities were high for all conditions and procedures, as were NPVs. The observed patterns were similar for the individual hospitals (Table 2).

**Table 1:** Reliability of reporting of bleeding-related conditions in coded hospital data across two tertiary hospitals, New South Wales, 2011–2015, compared to obstetric data (ObstetriX) as the reference standard

	ObstetriX (%)	Coded hospital data (%)	Both	Sensitivity (95% CI)	Specificity (95% CI)	PPV (95% CI)	NPV (95% CI)
Anaemia (nutritional or haemolytic)	1530 (4.2)	1425 (4.0)	206	13.5 (11.8–15.3)	96.5 (96.3–96.7)	14.5 (12.7–16.4)	96.2 (96.0–96.4)
Nutritional anaemias	1412 (3.9)	308 (0.9)	99	7.0 (5.7– 8.5)	99.4 (99.3–99.5)	32.1 (27.0–37.7)	96.3 (96.1–96.5)
Haemolytic anaemias	140 (0.4)	64 (0.2)	23	16.4 (10.7–23.6)	99.9 (99.8–99.9)	35.9 (24.3–48.9)	99.7 (99.6–99.7)
Iron deficiency anaemia	1341 (3.7)	1415 (3.9)	170	12.7 (10.9–14.6)	96.4 (96.2–96.6)	12.0 (10.4–13.8)	96.6 (96.4–96.8)
Anaemia (Hb < 110 g/L) in pregnancy	6819 (19.4)	1400 (3.9)	534	7.8 (7.2–8.5)	96.9 (96.7–97.1)	38.1 (35.6–40.8)	81.4 (81.0–81.8)
B12 deficiency anaemia	113 (0.3)	14 (0.0)	5	4.4 (1.5–10.0)	100.0 (100.0–100.0)	35.7 (12.8–64.9)	99.7 (99.6–99.8)
Any bleeding before birth^a^	985 (2.7)	1128 (3.1)	641	65.1 (62.0– 68.1)	98.6 (98.5– 98.7)	56.8 (53.9–59.7)	99.0 (98.9–99.1)
Antepartum haemorrhage	798 (2.2)	874 (2.4)	449	56.3 (52.7–59.7)	98.8 (98.7–98.9)	51.4 (48.0–54.7)	99.0 (98.9–99.1)
Intrapartum haemorrhage	155 (0.4)	130 (0.4)	65	41.9 (34.1–50.1)	99.8 (99.8–99.9)	50.0 (41.1–58.9)	99.8 (99.7–99.8)
Postpartum haemorrhage (PPH)	4330 (12.0)	4001 (11.1)	3585	82.8 (81.6–83.9)	98.7 (98.6–98.8)	89.6 (88.6–90.5)	97.7 (97.5–97.8)
PPH following caesarean section	1158 (10.7)	902 (8.3)	741	64.0 (61.2–66.7)	98.3 (98.1–98.6)	82.2 (79.5–84.6)	95.8 (95.4–96.2)
PPH following vaginal birth	3170 (12.6)	3097 (11.4)	2842	89.7 (88.5–90.7)	98.8 (98.7–99.0)	91.8 (90.7–92.7)	98.5 (98.4–98.7)
Red blood cell transfusion	575 (1.6)	681 (1.9)	531	92.4 (89.9–94.4)	99.6 (99.5–99.6)	78.0 (74.7–81.0)	99.9 (99.8–99.9)
Placental abruption	126 (0.3)	150 (0.4)	86	68.3 (59.4–76.3)	99.8 (99.8–99.9)	57.3 (49.0–65.4)	99.9 (99.9–99.9)
Placenta praevia	337 (0.9)	379 (1.1)	274	81.3 (76.7–85.3)	99.7 (99.6–99.8)	72.3 (67.5–76.7)	99.8 (99.8–99.9)
Placenta accreta	22 (0.1)	74 (0.2)	15	68.2 (45.1–86.1)	99.8 (99.8–99.9)	20.3 (11.8–31.2)	100.0 (100.0–100.0)
Thalassaemia	751 (2.1)	23 (0.1)	19	2.5 (1.5–3.9)	99.9 (99.9–100.0)	82.6 (61.2–95.1)	98.0 (97.8–98.1)
Platelet disorders	137 (0.4)	222 (0.6)	34	24.8 (17.8–32.9)	99.5 (99.4–99.55)	15.3 (10.9–20.7)	99.7 (99.7–99.8)
Coagulation disorders	191 (0.5)	87 (0.2)	22	11.5 (7.4–16.9)	99.8 (99.8–99.86)	25.3 (16.6–35.8)	99.5 (99.5–99.6)
Hysterectomy	25 (0.1)	29 (0.1)	24	96.0 (79.7–99.9)	99.9 (99.9–100.0)	82.8 (64.2–94.2)	100.0 (100.0–100.0)

*Hb* haemoglobin, derived from pathology in obstetrics data

^a^Antepartum haemorrhage, intrapartum haemorrhage, or placental abruption

**Table 2:** Consistency of reporting of anaemia and blood related disorders in coded hospital data compared to obstetric data from two tertiary hospitals, New South Wales, 2011–2015, by hospital

	Hospital one		Hospital two		
	Sensitivity (95% CI)	Specificity (95% CI)	Sensitivity (95% CI)		Specificity (95% CI)
Anaemia (nutritional or haemolytic)	10.8 (8.3–13.7)	96.8 (96.5–97.2)	14.9 (12.8–17.3)		96.3 (96.1–96.5)
Nutritional anaemia	5.7 (3.9–8.1)	99.5 (99.3–99.6)	7.7 (6.1–9.7)		99.4 (99.2–99.5)
Haemolytic anaemia	25.0 (12.1–42.2)	99.9 (99.8–99.9)	13.5 (7.6–21.6)		99.9 (99.8–99.9)
Iron deficiency anaemia	9.3 (6.9–12.3)	96.8 (96.4–97.1)	14.6 (12.3–17.2)		96.2 (96.0–96.5)
Anaemia (Hb < 110 g/L) in pregnancy	7.3 (6.2–8.6)	97.3 (96.9–97.6)	8.0 (7.3–8.8)		96.8 (96.5–97.0)
B12 deficiency anaemia	8.3 (1.0–27.0)	100.0 (99.9, 100.0)	3.4 (0.7–9.5)		100.0 (100.0, 100.0)
Any bleeding before birth	70.0 (65.5–74.2)	97.6 (97.3–97.9)	61.0 (56.8–65.2)		99.1 (99.0–99.2)
Antepartum haemorrhage	59.8 (54.6–64.8)	97.9 (97.6–98.1)	53.3 (48.4–58.1)		99.2 (99.1–99.3)
Intrapartum haemorrhage	38.3 (27.7–49.7)	99.7 (99.6–99.8)	45.9 (34.3–57.9)		99.9 (99.8–99.9)
Postpartum haemorrhage (PPH)	78.3 (76.4–80.1)	98.7 (98.5–99.0)	86.4 (85.0–87.8)		98.7 (98.5–98.8)
PPH following caesarean section	59.4 (55.5–63.1)	99.1 (98.7–99.4)	70.3 (66.0–74.3)		98.0 (97.6–98.3)
PPH following vaginal birth	88.2 (86.3–90.0)	98.5 (98.2–98.8)	90.6 (89.2–91.9)		99.0 (98.8–99.1)
Red Blood cell transfusion	92.0 (86.1–95.9)	99.8 (99.7–99.9)	92.5 (89.6–94.8)		99.5 (99.4–99.6)
Placental abruption	70.8 (55.9–83.0)	99.7 (99.6–99.8)	66.7 (55.1–76.9)		99.9 (99.8–99.9)
Placenta praevia	79.2 (69.7–86.8)	99.6 (99.5–99.7)	82.2 (76.7–86.8)		99.7 (99.7–99.8)
Placenta accreta	60.0 (14.7–94.7)	99.7 (99.5–99.8)	70.6 (44.0–89.7)		99.9 (99.9–99.9)
Thalassaemia	4.7 (2.1–9.1)	100.0 (100.0, 100.0)	1.9 (0.9–3.4)		100.0 (100.0, 100.0)
Platelet disorders	28.3 (16.0–43.5)	99.4 (99.2–99.5)	23.1 (14.9–33.1)		99.5 (99.4–99.6)
Coagulation disorders	14.0 (7.4–23.1)	99.8 (99.7–99.9)	9.5 (4.7–16.8)		99.8 (99.8–99.9)
Hysterectomy	100.0 (59.0, 100.0)	100.0 (99.9, 100.0)	94.4 (72.7–99.9)		100.0 (100.0, 100.0)

*Hb* haemoglobin, derived from pathology in ObstetriX data

Rates of diagnoses were similar across the two data sources, with the exception of anaemia (haemoglobin < 110 g/L) in pregnancy, B12 deficiency anaemia and coagulation disorders (Table 1). For anaemia, platelet disorders, coagulation disorders, and to a lesser extent placenta accreta, numbers were similar between the databases, however there was a low level of overlap, with different women identified in each source.

Rates of diagnoses and procedures were similar between hospitals, with the exception of postpartum haemorrhage (PPH), antepartum haemorrhage (APH) and nutritional anaemia, which were higher at Hospital One (Additional file 1: Table 2).

Sensitivity, specificity, PPV and NPV for reporting of anaemia were similar for nulliparous and parous women (Table 3). Sensitivity and PPV for anaemia tended to increase by year of birth, and were higher for hospital medical, private obstetrician or general practitioner-focussed models of care compared to midwife-centred care. Sensitivity tended to increase, with decreases in specificity and NPV, where women received a blood transfusion compared to where they did not, except for intrapartum haemorrhage (IPH) and APH, where transfusion was associated with decreased sensitivity.

**Table 3:** Consistency of reporting of anaemia and blood related disorders in coded hospital data compared to obstetric data (ObstetriX) from two tertiary hospitals, New South Wales, 2011–2015, by pregnancy characteristics

Condition	Characteristic	Value	Source of identification of condition					NPV (95% CI)
			ObstetriX data	Coded hospital data	Sensitivity (95% CI)	Specificity (95% CI)	PPV (95% CI)	
Anaemia (nutritional or haemolytic)	Overall		1530	1425	13.5 (11.8–15.3)	96.5 (96.3–96.7)	14.5 (12.7–16.4)	96.2 (96.0–96.4)
	Parity	Nulliparous	758	809	13.2 (10.9–15.8)	95.6 (95.3–95.9)	12.4 (10.2–14.8)	95.9 (95.6–96.2)
		Parous	766	607	13.7 (11.4–16.3)	97.3 (97.0–97.5)	17.3 (14.4–20.5)	96.4 (96.1–96.7)
	Birth year	2011	262	176	8.0 (5.0–12.0)	97.7 (97.3–98.0)	11.9 (7.5–17.7)	96.4 (96.0–96.9)
		2012	263	146	7.6 (4.7–11.5)	98.2 (97.8–98.5)	13.7 (8.6–20.4)	96.5 (96.1–96.9)
		2013	281	259	11.7 (8.2–16.1)	96.7 (96.2–97.1)	12.7 (8.9–17.4)	96.3 (95.9–96.8)
		2014	355	455	22.0 (17.8–26.6)	94.8 (94.3–95.3)	17.1 (13.8–20.9)	96.1 (95.6–96.6)
		2015	369	389	14.6 (11.2–18.7)	95.2 (94.7–95.7)	13.9 (10.6–17.7)	95.5 (95.0–96.0)
	Model of care	Hospital medical ^a^	625	642	18.2 (15.3–21.5)	96.1 (95.7–96.4)	17.8 (14.9–20.9)	96.2 (95.8–96.5)
		Midwife ^a^	820	687	9.5 (7.6–11.7)	96.6 (96.3–96.9)	11.4 (9.1–14.0)	95.9 (95.6–96.2)
		Private obstetrician or GP only	81	91	17.3 (9.8–27.3)	97.5 (96.9–98.0)	15.4 (8.7–24.5)	97.8 (97.3–98.3)
	Transfusion status	Transfused	50	382	82.0 (68.6–91.4)	35.0 (31.0–39.3)	10.7 (7.8–14.3)	95.3 (91.3–97.8)
		Not transfused	1480	1043	11.1 (9.6–12.9)	97.4 (97.2–97.6)	15.8 (13.7–18.2)	96.2 (96.0–96.4)
PPH	Overall		4330	4001	82.8 (81.6–83.9)	98.7 (98.6–98.8)	89.6 (88.6–90.5)	97.7 (97.5–97.8)
	Transfusion status	Transfused	482	456	88.8 (85.6–91.5)	69.9 (59.5–79.0)	93.9 (91.2–95.9)	54.6 (45.2–63.8)
		Not transfused	3848	3545	82.0 (80.8–83.2)	98.8 (98.6–98.9)	89.1 (88.0–90.1)	97.8 (97.7–98.0)
IPH	Overall		155	130	41.9 (34.1–50.1)	99.8 (99.8–99.9)	50.0 (41.1–58.9)	99.7 (99.7–99.8)
	Transfusion status	Transfused	12	13	25.0 (5.5–57.2)	98.2 (96.8–99.1)	23.1 (5.0–53.8)	98.4 (97.0–99.3)
		Not transfused	143	117	43.4 (35.1–51.9)	99.8 (99.8–99.9)	53.0 (43.5–62.3)	99.8 (99.7–99.8)
APH	Overall		798	874	56.3 (52.7–59.7)	98.8 (98.7–98.9)	51.4 (48.0–54.7)	99.0 (98.9–99.1)
	Transfusion status	Transfused	46	30	34.8 (21.4–50.2)	97.4 (95.6–98.5)	53.3 (34.3–71.7)	94.5 (92.2–96.3)
		Not transfused	752	844	57.6 (54.0–61.1)	98.8 (98.7–98.9)	51.3 (47.9–54.7)	99.1 (99.0–99.2)
Thalassaemia	Overall		751	23	2.5 (1.5–3.9)	100.0 (100.0–100.0)	82.6 (61.2–95.0)	98.0 (97.8–98.1)
	Transfusion status	Transfused	25	3	12.0 (2.5–31.2)	100.0 (99.3–100.0)	100.0 (29.2, 100.0)	96.2 (94.2–97.6)
		Not transfused	726	20	2.2 (1.3–3.6)	100.0 (100. 100.0)	80.0 (56.3–94.3)	98.0 (97.8–98.1)

*GP* general practitioner

^a^Including shared GP

Potential misclassification was identified, with 32.2% of the 58.1% whose IPH was not identified in the hospital data reported with PPH (29 of the 90‬ missed cases), compared to 16.9% (11 of 65) among those whose IPH was reported in the hospital data. In comparison, among those whose PPH was not identified in the hospital data (745 of 4330), 3.0% were recorded with IPH (22 of 745), compared to 0.6% among those whose PPH reported in ObstetriX (20 of 3565‬).

## Discussion

All types of anaemia were under-ascertained in ICD-coded hospital data, with a high proportion of cases reported in the hospital data and not in obstetric data. Using combined categories for anaemia did not meaningfully improve sensitivity or PPV. Thalassaemia, platelet disorders, and coagulation disorders were similarly underreported. Reliability for APH and IPH were moderate, and using a broad category for any bleeding before birth improved sensitivity and PPV. Postpartum haemorrhage, transfusion, placental complications (including accreta, praevia, and abruption), and hysterectomy, were reported with moderate to high consistency in the obstetric data. Compared with previous studies, we found better reporting of hysterectomy [13] and PPH [13], but poorer reporting for APH [13], coagulation disorders [11], placenta praevia [12, 13], haemolytic anaemia [11], and placenta accreta [13]. Accuracy was similar to previous studies for transfusion [13, 14], placental abruption [12, 13] and nutritional anaemia [11].

Consistency of reporting of anaemia improved with time and where antenatal care was provided by a hospital medical team, private obstetrician or general practitioner, compared to midwife-centred care. This may be reflective of a lack of an agreed definition of anaemia for pregnant women, amid discrepancies between the National Blood Authority and World Health Organisation definitions [27]. Having a transfusion affected reliability of reporting, with anaemia showing higher sensitivity for patients who received a blood transfusion, however this reduced specificity and PPV remained low. Having a transfusion made little difference to sensitivity for PPH, although it was associated with a reduction in specificity, and transfusion was associated with reduced sensitivity for APH and IPH. Having a transfusion slightly increased sensitivity for thalassaemia, although it remained poorly reported among that group. While data on bleeding severity were not available, previous studies have shown that the likelihood that haemorrhage or transfusion are reported in hospital data increases with higher severity [13, 14]

The inconsistent reporting of anaemia and blood disorders reflects the difficulties in capturing chronic but minor conditions not affecting the current admission. Serious, persistent conditions that are more likely to affect the hospital admission, including diabetes and hypertension, have demonstrated better correlation between datasets [28]. The differences in timing of data collection likely affected consistency, as issues may arise or resolve between early pregnancy and birth, particularly for fluctuating conditions such as iron-deficiency anaemia. Indeed, a recent study of the same study population and datasets as examined here found that 38% of women recorded with low haemoglobin (< 110 g/L) in the first 20 weeks of pregnancy had their haemoglobin level restored (110 g/L or higher) after 20 weeks gestation, with 38% not restored and 24% not recorded, likely reflecting good antenatal care and the recent emphasis on treating anaemia as a pillar of patient blood management [29]. The increase in anaemia prevalence, from 3.9% based on clinical diagnosis in the hospital data compared to 19.4% according to pathology results, however, highlights discrepancies between the language clinicians use in the medical notes and the language required for clinical coding, such that Hb = 69 g/L cannot be interpreted as anaemia by a coder if the words ‘anaemia’ or ‘low Hb’ are not written in the notes [17]. Additionally, a code may not be assigned for a queried diagnosis. Regarding obstetric haemorrhage, while bleeding may occur before labour (APH), during labour but before the birth (IPH) or following birth (PPH), and should be recorded as such, practically, conflation of IPH and PPH is not uncommon and it is the total blood loss rather than the exact timing of bleeding that guides the clinical response. Progressive loss during labour may not be separated into discrete time periods in the documentation or data entry, particularly when IPH occurs close to the time of birth. Hence, a composite measure may be more useful.

There was a lack of overlap in patients recorded with anaemia, platelet disorders, coagulation disorders, and placenta accreta in the two databases. For anaemia, this may be related to the timing of data collection, as discussed above, which may contribute to the high proportion of “false” cases in the hospital data. While one of the strengths of ObstetriX is that it uses systematic pre-specified fields, making omissions less likely than for hospital data, it is likely that for some conditions, obstetric data are incomplete. For transfusion, hospital data are likely more complete than obstetric data given that blood must be dispensed and recorded in patient notes. Previous studies have shown high reliability for transfusion in hospital data compared to blood pack information from transfusion laboratories [14]. For placenta accreta, hospital data are likely more accurate, given that coding considers placental histopathology and procedures such as manual removal of placenta. Obstetric data are largely self-reported, which may contain inaccuracies [24]. Further, data are entered into ObstetriX by midwives, with accuracy sometimes sacrificed when personnel are busy providing clinical care, and the person entering the data was generally not present for the entire episode of care. In light of our findings, we suggest that hospital data may be a more appropriate source for identifying transfusion than obstetric data, while for the remaining conditions, using both data sources to identify cases, possibly corrected with capture-recapture models [30], is advisable where possible for improving ascertainment. This supports recommendations made elsewhere [18, 31].

Slight differences in the rates of diagnoses between hospitals likely relate to the different demographic compositions, with Hospital Two tending to have a younger obstetric population with a different ethnic and comorbidity mix. However, the overall consistency between the two hospitals suggests that the results hold across different socioeconomic and ethnic patient populations, locations, and facilities.

## Conclusion

We found poor reporting of anaemia and blood disorders. Reporting of ante- and intrapartum haemorrhage and placental complications were moderately well reported, while hysterectomy, transfusion and PPH showed high consistency between datasets. Procedures were better reported than conditions, and conditions occurring around the time of birth, such as PPH, were better reported than pre-existing conditions, such as blood disorders. Caution should be exercised in the use of hospital data for studies of anaemia and blood disorders, given both the high rates of missed cases and cases unconfirmed in obstetric data, and other sources of data should be sought where possible. These findings are likely to be reasonably generalizable, given that ICD-10 is widely used [31, 32], and previous studies have shown similar accuracy between hospital data from NSW and elsewhere [33].

## Limitations

Obstetric data is an imperfect reference standard. The data are collected at different times, which may have affected the results as issues may arise or resolve in early pregnancy and birth. ObstetriX data are largely self-reported, which may be inaccurate [24], and in some cases may be less complete than hospital data. The implications of these limitations are discussed in relation to the findings above. Data were available from two hospitals only.

## Supplementary Information

**Additional file 1**: **Table 1**. International Classification of Diseases, Tenth Edition, Australian Modification (ICD-10-AM) diagnostic and Australian Classification of Health Interventions, Eighth Edition (ACHI) procedure codes for anaemia and bleeding disorders used in this study. **Table 2**. Rates of diagnoses and procedures identified by hospital and data source, New South Wales, 2011-2015.

## Data Availability

The data that support the findings of this study are available from Northern Sydney Local Health District and Western Sydney Local Health District, but privacy and confidentiality restrictions apply to the availability of these data, which were provided following ethical approval for the current study, but are not publicly available. Data are however directly available from the Northern Sydney and Western Sydney Local Health Districts upon request and ethical approval.

## Abbreviations

ICD: International Classification of Diseases
PPV: Positive predictive value
NPV: Negative predictive value
PPH: Postpartum haemorrhage
IPH: Intrapartum haemorrhage
APH: Antepartum haemorrhage
NSW: New South Wales
