# Supplementary material for: Validation of anaemia, haemorrhage and blood disorder reporting in hospital data in New South Wales, Australia
Source: BMC Res Notes. 2021 May 4;14:167. doi: 10.1186/s13104-021-05584-x (PMC8094525; doi:10.1186/s13104-021-05584-x)
Supplement: Supplementary file 1 — Additional file 1: Table 1. International Classification of Diseases, Tenth Edition, Australian Modification (ICD-10-AM) diagnostic and Australian Classification of Health Interventions, Eighth Edition (ACHI) procedure codes for anaemia and bleeding disorders used in this study. Table 2. Rates of diagnoses and procedures identified by hospital and data source, New South Wales, 2011-2015. [file 13104_2021_5584_MOESM1_ESM.docx]

**Supplementary Table 1.** International Classification of Diseases, Tenth Edition, Australian Modification (ICD-10-AM) diagnostic and Australian Classification of Health Interventions, Eighth Edition (ACHI) procedure codes for anaemia and bleeding disorders used in this study

| **Condition** | **Diagnostic code** | **Procedure code** |
| --- | --- | --- |
| Antepartum haemorrhage | O46 |  |
| Placental abruption | O45 |  |
| Placenta praevia | O44.1, O44.0 |  |
| Placenta accreta^1^ | O43.2 |  |
| Any bleeding before birth | O45, O46, O67, O72 |  |
| Intrapartum haemorrhage | O67 |  |
| Postpartum haemorrhage (PPH) | O72 |  |
| PPH following caesarean section | O72 | With 16520 |
| PPH following vaginal birth | O72 | Without 16520 |
| Red blood cell transfusion | 13706-01, 13706-02, 92206-00, 413852010, 428230014, Z51.3 |  |
| Anaemia (nutritional or haemolytic)^2^ | D50–D65, 0.99.0 |  |
| Iron deficiency anaemia | D50, O99.0 ,E61.1 |  |
| Anaemia (Hb<110g/L) in pregnancy^3^ | D50, O99.0, E61.1, D62 |  |
| Nutritional anaemias | D50, D51, D52, D53 |  |
| Haemolytic anaemias | D55, D56, D57, D58, D59 |  |
| B12 deficiency anaemia | D51, D52, E53.8 |  |
| Thalassaemia | D56.0, D56.1 |  |
| Platelet disorders | D69 |  |
| Coagulation disorders | D66, D67, D68 |  |
| Hysterectomy |  | 35653, 35661, 35664, 35667, 35670, 90448, 35657, 35664, 35667, 35673, 35750, 35753, 35756, 35756, Hysterectomy |

^1^ reported in two variables in the obstetrics data: third stage complications or indication for caesarean

^2^ any type of anaemia specified in categorical anaemia dropdown variable in obstetrics data

^3^ Hb = haemoglobin, based on pathology results in ObstetriX

**Supplementary Table 2.** Rates of diagnoses and procedures identified by hospital and data source, New South Wales, 2011-2015.

| Variable | Hospital One-ObstetriX  n(%) | Hospital One-coded hospital data n(%) | Hospital Two-ObstetriX n(%) | Hospital Two- coded hospital data n(%) |
| --- | --- | --- | --- | --- |
| Anaemia (nutritional or haemolytic) | 539 (4.7) | 401 (3.5) | 991 (4.0) | 1024 (4.2) |
| Nutritional anaemias | 507 (4.5) | 905 (3.7) | 84 (0.7) | 224 (0.9) |
| Haemolytic anaemias | 36 (0.3) | 104 (0.4) | 23 (0.2) | 41 (0.2) |
| Iron deficiency anaemia | 492 (4.3) | 397 (3.5) | 849 (3.4) | 1018 (4.1) |
| Anaemia (Hb<110g/L) in pregnancy | 1904 (16.7) | 396 (3.5) | 4915 (19.9) | 1020 (4.1) |
| B12 deficiency anaemia | 24 (0.2) | 6 (0.1) | 89 (0.4) | 8 (0.0) |
| Any bleeding before birth | 446 (3.9) | 539 (2.2) | 577 (5.1) | 551 (2.2) |
| Antepartum haemorrhage | 368 (3.2) | 456 (4.0) | 430 (1.7) | 418 (1.7) |
| Intrapartum haemorrhage | 81 (0.7) | 63 (0.6) | 74 (0.3) | 67 (0.3) |
| Postpartum haemorrhage (PPH) | 1937 (17.0) | 1636 (14.4) | 2393 (9.7) | 2365 (9.6) |
| PPH following caesarean section | 667 (5.9) | 425 (3.7) | 491 (2.0) | 479 (1.9) |
| PPH following vaginal birth | 1270 (11.2) | 1211 (10.6) | 1902 (7.7) | 1886 (7.6) |
| Red blood cell transfusion | 137 (1.2) | 149 (1.3) | 438 (1.8) | 532 (2.2) |
| Placental abruption | 48 (0.4) | 70 (0.6) | 78 (0.3) | 80 (0.3) |
| Placenta praevia | 96 (0.8) | 117 (1.0) | 241 (1.0) | 262 (1.1) |
| Placenta accreta | 5 (0.0) | 41 (0.4) | 17 (0.1) | 33 (0.1) |
| Thalassaemia | 169 (1.5) | 8 (0.1) | 582 (2.4) | 15 (0.1) |
| Platelet disorders | 46 (0.4) | 84 (0.7) | 91 (0.4) | 139 (0.6) |
| Coagulation disorders | 86 (0.8) | 35 (0.3) | 105 (0.4) | 52 (0.2) |
| Hysterectomy | 7 (0.1) | 9 (0.1) | 18 (0.1) | 20 (0.1) |

^1^ Hb = haemoglobin, derived from pathology in obstetrics data
